# Using operating room turnover time by anesthesia trainee level to assess improving systems-based practice milestones

**DOI:** 10.1186/s12909-018-1409-6

**Published:** 2018-12-05

**Authors:** Christopher Ryan Hoffman, Michael Stuart Green, Jasmine Liu, Usama Iqbal, Kirtanaa Voralu

**Affiliations:** 1 Department of Anesthesiology & Perioperative Medicine, Drexel University College of Medicine, Hahnemann University Hospital, 245 N. 15th Street, Suite 7502, MS 310, Philadelphia, PA 19102 USA; 2 Houston Methodist Hospital, Houston, TX USA

**Keywords:** Health resources, Resource allocation, Graduate medical education, Educational measurement, Anesthesia

## Abstract

**Background:**

Operating room (OR) metrics are frequently cited when optimizing cost efficacy and quality of care (Weiss et al, Characteristics of operating room procedures in U.S. hospitals, 2011: Statistical brief #170, 2013; Macario A, Anesthesiology 105:237-240, 2006; Childers et al, JAMA Surg 153:e176233, 2018). Little has been reported to evaluate how anesthesia trainees change anesthesia-related efficiencies in the OR. Statistical correlation may demonstrate awareness and implementation of efficient systems-based practice.

**Methods:**

Utilizing computerized OR information systems, specific data regarding anesthesia controlled turnover times were collected (546 data points) over the course of 4 months. The type of surgery performed, patient’s American Society of Anesthesiologists (ASA) physical status and OR turnover times were compared for clinical anesthesia (CA) trainee levels CA1, CA2, CA3 and CRNAs. Standard descriptive statistics were computed. Analysis of variance (ANOVA) was performed to compare the average turnover time.

**Results:**

Average OR turnover time was 31 min ranging from 8 to 60 min. There was a significant difference between the OR turnover time of CA-1 (32 min) compared to CA-3 (29 min) (*p =* 0.017) and CA-1 compared to CRNA (30 min) (*p* = 0.016). OR turnover time was significantly shorter in CA-3 and CRNA. The analysis showed no differences between OR turnover time of ASA categories.

**Conclusions:**

These findings posit that trainees improve efficiency over time, but that education may for a time come at the expense of productivity. This trend may demonstrate a more profound understanding and mastery of a learner progressing in the graduate medical education system. This interplay plays a key role in clinical and academic shared success.

## Background

The need to optimize health care quality is substantial. Improvement is facilitated when providers regularly review performance, design interventions, and create teams to implement change. Operating rooms (ORs) are one of the most resource-consuming areas of a hospital to provide surgical care. A recent release from the US Healthcare Cost and Utilization Project found that the cost of a hospital stay after operative procedures was 2.5 times more expensive than that of a hospitalized patient not requiring such procedures [1]. Optimal allocation of OR resources is a major priority to ensure cost-efficiency. Excess staffing, case start delays/cancelations, case duration, and case turnover are some contributors to resource allocation [2]. Time is the most precious resource of an OR. Cost attributable to fixed overhead to utilize an OR is an estimated $20 per minute. When factoring in physician and nursing staff, hospitals charge between $30–40 per minute depending on the complexity of the case [3]. Delayed case start times, prolonged turnover times, or equipment issues can hinder OR efficiency.

Training anesthesia residents might increase process times, negatively influence OR efficiency, and increase waiting time for surgeons [4]. Teaching cases are associated with an increased anesthesia induction time by a mean of 4.5 min (standard deviation 3.2 min) [5]. The introduction of new anesthesia residents causes a statistically significant effect on induction, emergence, and turnover times [6]. Other data suggests that decreases in anesthesia controlled time doesn’t save enough time to increase scheduled surgical cases [7].

Although qualitative factors like professional satisfaction among surgeons and OR staff are a relevant priority, educational requisites are another aspect to consider [8]. One of the six core competencies dictated by The Accreditation Council for Graduate Medical Education (ACGME) is systems-based practice and “using system resources to facilitate cost-effective and safe non-subspecialty anesthesia care” [9]. Studies correlating training level and quality of care are scarce. Some show heterogeneous results with some inefficiencies found in specific procedure times or new exposure to subspecialty cases [10, 11]. Efforts to improve cost efficiency in the academic setting may threaten thorough training in a fostering environment [12]. Ensuring efficiency for trainees is a challenging blend of hospital cost, ACGME core competency, and effective education. Previously, Eappen et al. concluded that OR metrics across three two-week periods in a single resident training year concluded “no clinically or economically meaningful adverse effect on the anesthesia-controlled time component of operating room efficiency” [6]. In this study, we hypothesized that turnover times are correlated with the level of training for anesthesiology residents. We aim to look at a broader amount of trainee levels over a larger span of time to observe for competency.

## Methods

### Data collection

Permission to retrospectively review OR case data was granted by the Institutional Review Board of our facility (Protocol #1701005145). Turnover times were collected from patients 18–75 years old undergoing common ambulatory procedures under general anesthesia or monitored anesthesia care (MAC). Procedures that were delayed for external reasons (e.g. subsequent patient did not arrive, admission/post anesthesia care unit (PACU) delay, pending diagnostic test, surgeon or OR staff delay, etc.) were excluded. Turnover times were included only when staff remained the same between cases. Cases in which CA-1’s were in the initial 8 weeks of orientation were excluded. Procedures with unique requirements that may skew results were excluded (e.g. emergencies, cases requiring preprocedure nerve blocks/vascular access/neuromonitors, cases transported directly to ICU). High-acuity or subspecialty cases unevenly assigned to upper-level residents were excluded to ensure each group of cases were similar. A sample size of 100 data points per group was determined. Past cases were reviewed one day at a time into the past until all the sample sizes were achieved, yielding a final data set of 546 data points.

### Turnover time

Turnover time is defined as the time from the prior patient exiting the OR to the next patient entering the OR. We evaluated four groups: clinical anesthesia (CA) residents in the first, second, and third years, and certified registered nurse anesthetists (CRNA). We measured the OR turnover time in the CA-1, CA-2, and CA-3 resident levels and observed any difference in total or improved times among the residents or between residents and CRNAs.

Basic demographic data collected included the patient age, gender, and weight. Type and duration of surgery were also recorded. We did not attempt to control or modify the specific anesthetic techniques the groups used. Since it was a retrospective study, the nurse anesthetists and residents involved in the care of study patients were not aware of the study or of any specific parameters being measured. Thus, each anesthesiology trainee level developed and executed an anesthetic plan according to his/her usual practice.

### Statistical analysis

Data analysis was performed with IBM SPSS (IBM Corp. Released 2016. IBM SPSS Statistics for Windows, Version 24.0. Armonk, NY: IBM Corp). Number of cases and percentages, mean, standard deviation (SD), median and range were obtained for OR turnover time according to ASA of previous OR case patient, ASA of next OR case patient and anesthesia trainee level. Analysis of variance (ANOVA) was performed to compare the average turnover time. Post-hoc analysis using Least Significant Difference (LSD) test were done to identify significant differences between the OR turnover time.

## Results

A total of 546 data points were compared in this study. Data points were studied by CA-1 (*n* = 177), CA-2 (*n* = 100), CA-3 (*n* = 99), and CRNA (*n* = 170) over 4 months. The overall average OR turnover time was 31 min ranging from 8 to 60 min. Mean OR turnover time was 32 min for CA-1 and CA-2 while 30 min for CRNAs and 29 min for CA-3. OR turnover time was significantly shorter in CA-3 and CRNA. The analysis showed no differences in OR turnover time among ASA categories (Tables 1 and 2).

**Table 1:** Summary of OR turnover time according to ASA and anesthesia trainee level at Hahnemann University Hospital

	Number of OR data points (n, %)	OR turnover time (minutes)	
		Mean (SD)	Median (Range)
Overall	546 (100)	31 (10)	30 (8–60)
ASA of previous OR case patient			
I	35 (6.4)	30 (11)	27 (14–56)
II	265 (48.7)	31 (10)	30 (9–60)
III	242 (44.5)	30 (10)	29 (8–57)
IV	2 (0.4)	28 (11)	28 (20–35)
ASA of next OR case patient			
I	27 (5.0)	29 (8)	27 (19–49)
II	241 (44.5)	31 (10)	29 (10–60)
III	272 (50.3)	31 (10)	30 (8–57)
IV	1 (0.2)	24	24
Anesthesia trainee level			
CA-1	177 (32.4)	32 (10)	31 (10–58)
CA-2	100 (18.3)	32 (10)	30 (9–60)
CA-3	99 (18.1)	29 (10)	28 (8–53)
CRNA	170 (31.2)	30 (9)	29 (12–55)

**Table 2:** Comparison OR turnover time according to ASA and anesthesia trainee level at Hahnemann University Hospital

OR turnover time (minutes)	N	Mean (SD)	95% Confidence Interval of mean	F-test (df)	*p-*value
ASA of previous OR case patient					
I	35	30 (11)	26, 33	0.8 (3, 540)	0.503
II	365	31 (10)	30, 33		
III	242	30 (10)	29, 32		
IV	2	28 (11)	−68, 123		
ASA of next OR case patient					
I	27	29 (8)	26, 32	0.6 (3, 537)	0.590
II	241	30 (10)	29, 32		
III	272	31 (10)	30, 32		
IV	1	24	–		
Anesthesia trainee level					
CA-1	177	32 (10)	31, 34	3.0 (3, 542)	0.030
CA-2	100	32 (10)	30, 34		
CA-3	99	29 (10)	27, 31		
CRNA	170	30 (9)	28, 31		

A post-hoc analysis showed significant difference between the OR turnover time of CA-1 compared to CA-3 with significant *p* value of 0.017 and CA-1 compared to CRNA with significant p value of 0.016. 95% confidence interval of mean difference was 0.54–5.44 and 0.48–4.67 respectively (Table 3).

**Table 3:** Post-hoc analysis between OR turnover time and anesthesia trainee level Hahnemann University Hospital

OR turnover time (minutes)	Mean Difference (Standard Error)	95% Confidence Interval of Mean Difference	*p*-value
Anesthesia trainee level			
CA-1 vs CA-3	2.99 (1.25)	0.54, 5.44	0.017
CA-1 vs CRNA	2.57 (1.07)	0.48, 4.67	0.016

## Discussion

Our study shows that by the end of training there is a statistically significant reduction in turnover time. Even though this amounts to 2–3 min, this is a 10% reduction in turnover time. A 10% improvement in any heavily valued performance metric warrants attention. This percentage may not have much opportunity for further improvement as general OR turnover is the same across most surgical cases. With multiple cases throughout the day this can accumulate to a sizeable amount of time. It may or may not be enough to fit additional OR cases but can ensure that OR staffing and scheduled time remains properly utilized and avoids overtime expense [13, 14]. It is important for residents to appreciate achieving acceptable turnover times in order to perform competitively in future practices as well as establishing good rapport with staff and surgeons.

Prolonged turnover time can occur due to factors that are beyond our control. Our study tried to exclude external factors such as cases involving extra preparation, extremely sick patients, more complicated or prolonged surgeries, and delays due to non-anesthesiology team members. We removed the possibility of working faster under observation as our study is retrospective, so subjects were not aware times were being analyzed. Turnover time is when a patient exits the OR and the next patient enters the same OR [15]. These are recorded times which may not be properly recorded and don’t denote an exact timeline. Times could be rounded up or down. We did attempt to eliminate turnover data points if there were delays admitting patients into preoperative holding or the PACU. Variable time it takes to transition care to PACU nursing, including PACU nurse unavailability and lengthy paperwork completion, can affect how long it takes for a provider to return to the OR and prepare for subsequent cases. A better measure may be when a provider is done signing out their patient in PACU to when their next patient is taken to the same OR. A potential bias we were unable to avoid is that the study was done only at our program, where many of our patients have multiple comorbidities. It is possible that we could have seen statistically significant difference between ASA classes if patients had fewer or different comorbidities. Excluding specialty cases in an attempt to control data may also skew results. Nursing staffing may be increased and attending anesthesiologist staffing may be one-on-one in this scenario, potentially streamlining turnover efforts. Retrospectively reviewing high-dimensional data may present a challenge when utilizing standard computational and statistical techniques. A larger scale prospective review of many anesthesia providers caring for patients with different ranges in comorbidities may elicit different results.

The anesthesia provider is one facet of a multidimensional process, and other sources of inefficiency contribute to delays. Complicated or delayed OR cleaning and subsequent surgical/anesthesia equipment setup is not a reflection of the anesthesia provider but may be credited to their turnover time. Decreased turnover time may not be a reflection of the provider’s capability. Medical student, student nurse anesthetist, or anesthesia assistant contributions toward case turnover is unaccounted for with unpredictable impact. Supervising anesthesiologists may affect turnover time depending on incoming patient evaluation, consent, and intravenous cannulation while the previous case is still in progress. Other undocumented factors could affect times and yet would not be shown in the record.

Turnover time is regarded as a critical component in optimizing OR management. Improvement has been correlated with better utilization of resources, reduction in staffing costs and wasted resources, and patient/staff satisfaction. Much emphasis has been paid upon anesthesiology-related metrics and how anesthesia trainees affect them. Prior reporting demonstrates mixed results overall with no significant increase in time for turnover. Our study is novel in its stratification amongst different class years and how turnover time may be improved with every successive year. The goal in identifying an improving trend theoretically translates to resident improvement in future practices. Optimal OR suites are able to turnover cases in 25 min or less [2]. Our study shows that residents can make significant enough progress to reach such a goal, and that senior residents are not a definite hindrance to OR efficiency. This is consistent with prior literature reporting anesthesia emergence times improving with ongoing training. Reviewing other metrics across residency training years may yield similar results.

The ACGME views “facilitating cost-effective and safe anesthesia care” as part of the systems-based practice milestone to evaluate a resident competency. There is little objective consensus on how to evaluate this. The study’s intention of evaluating how resident training may affect cost efficacy in academic facilities also introduces the possibility of using OR metrics as a way to monitor progression. Given the amount of potential bias, this may not seem a practical nor an accurate reflection of the resident’s progress. OR metrics more reliant on the provider that improve with experience (e.g. induction or emergence times, auditing drugs and resources utilization) may be better indicators. The “business of anesthesia” involves a managerial skill-set apart from clinical aptitude and may require qualitative monitoring [16]. Despite hospital emphasis on efficient turnover and increasing profit, the goal remains the same: efficiency without sacrificing education or patient safety. With these results, we hope to conduct further studies to stratify how improvements can be observed in resident training. Using metrics that anesthesia residents may have little control over may inappropriately assess competency and incorrectly monitor cost efficacy implications.

## Conclusions

There was a significant difference between the OR turnover time of CA-1 compared to CA-3 (*p =* 0.017) and CA-1 compared to CRNA (*p* = 0.016). OR turnover time was significantly shorter in CA-3 and CRNA. The analysis showed no differences between OR turnover time of ASA categories. There is a statistically significant improvement and reduction in turnover time. Even though this amounts to just 2–3 min, this amounts to a 10% reduction in turnover time. These findings demonstrate that anesthesia residents become more efficient over time. The relative inefficiencies of a resident’s workflow at the beginning of training must be balanced by the education mission. These two points potentially have a substantial impact on optimizing education quality and cost efficacy in the healthcare system. Furthermore, what is not known is if this trend is simply a learning curve improved by experience, or a more profound understanding and mastery of a learner progressing in the graduate medical education system. How these competing priorities intertwine is pivotal to the discussions about the educational missions for health care institutions.

## Abbreviations

ACGME: Accreditation Council for Graduate Medical Education
ANOVA: Analysis of Variance
ASA: American Society of Anesthesiologists
CA: Clinical Anesthesia
CRNA: Certified Nurse Anesthetist
df: Degrees of Freedom
LSD: Least Significant Difference
MAC: Monitored Anesthesia Care
OR: Operating Room
PACU: Post Anesthesia Care Unit
SD: Standard Deviation
